# Norlichexanthone Reduces Virulence Gene Expression and Biofilm Formation in *Staphylococcus aureus*

**DOI:** 10.1371/journal.pone.0168305

**Published:** 2016-12-22

**Authors:** Mara Baldry, Anita Nielsen, Martin S. Bojer, Yu Zhao, Cathrine Friberg, Dan Ifrah, Nina Glasser Heede, Thomas O. Larsen, Hanne Frøkiær, Dorte Frees, Lixin Zhang, Huanqin Dai, Hanne Ingmer

**Affiliations:** 1 Department of Veterinary Disease Biology, Faculty of Health and Medical Sciences, University of Copenhagen, Frederiksberg C, Denmark; 2 Chinese Academy of Sciences Key Laboratory of Pathogenic Microbiology and Immunology, Institute of Microbiology, Beijing, China; 3 Center for Microbial Biotechnology, Department of Systems Biology, Technical University of Denmark, Kongens Lyngby, Denmark; Universitatsklinikum Hamburg-Eppendorf, GERMANY

## Abstract

*Staphylococcus aureus* is a serious human pathogen and antibiotic resistant, community-associated strains, such as the methicillin resistant *S*. *aureus* (MRSA) strain USA300, continue to spread. To avoid resistance, anti-virulence therapy has been proposed where toxicity is targeted rather than viability. Previously we have shown that norlichexanthone, a small non-reduced tricyclic polyketide produced by fungi and lichens, reduces expression of *hla* encoding α-hemolysin as well as the regulatory RNAIII of the *agr* quorum sensing system in *S*. *aureus* 8325–4. The aim of the present study was to further characterise the mode of action of norlichexanthone and its effect on biofilm formation. We find that norlichexanthone reduces expression of both *hla* and RNAIII also in strain USA300. Structurally, norlichexanthone resembles ω-hydroxyemodin that recently was shown to bind the *agr* two component response regulator, AgrA, which controls expression of RNAIII and the phenol soluble modulins responsible for human neutrophil killing. We show that norlichexanthone reduces *S*. *aureus* toxicity towards human neutrophils and interferes directly with AgrA binding to its DNA target. In contrast to ω-hydroxyemodin however, norlichexanthone reduces staphylococcal biofilm formation. Transcriptomic analysis revealed that genes regulated by the SaeRS two-component system are repressed by norlichexanthone when compared to untreated cells, an effect that was mitigated in strain Newman carrying a partially constitutive SaeRS system. Our data show that norlichexanthone treatment reduces expression of key virulence factors in CA-MRSA strain USA300 via AgrA binding and represses biofilm formation.

## Introduction

*Staphylococcus aureus* is a serious human bacterial pathogen that causes infections ranging from minor skin and soft tissue infections to osteomyelitis, sepsis and necrotizing pneumonia [1]. In the early 1990’s highly invasive community-associated methicillin resistant *S*. *aureus* (CA-MRSA) strains emerged such as USA300 [2,3]. This strain has now become one of the predominant CA-MRSA clones and it is evolving towards resistance against several antibiotic classes [4]. With the increase in problems associated with antimicrobial resistance, alternative treatments are being considered. One option is anti-virulence therapy where compounds are sought to reduce virulence rather than pathogen viability [5,6]. In *S*. *aureus*, the *agr* quorum sensing system controls expression of numerous virulence factors in response to cell density, including α-hemolysin and the phenol-soluble modulins (PSMs), that contribute particularly to the virulence of CA-MRSA [7]. The *agr* P2 transcript encodes a two-component signal transduction system composed of the response regulator, AgrA, and the sensor histidine kinase, AgrC that responds to secreted autoinducing peptide (AIP). AgrA controls expression of the divergently transcribed RNAIII expressed from the P3 promoter as well as of the PSMs [8,9]. Other two-component systems also modulate virulence including the auto-regulated SaeRS that responds to external stimuli such as pH, NaCl, sub-inhibitory concentrations of antimicrobial peptides and human skin fatty acids [10–12]. In addition to virulence, both *agr* and SaeRS influence biofilm formation in *S*. *aureus*, with *agr* acting through production of the PSMs [13] and SaeRS by repressing production of extracellular proteases degrading proteins of importance to biofilm formation [14].

Several natural compounds have been found to target virulence gene expression in *S*. *aureus* including the solonamides, which are cyclic peptides isolated from a marine Gram-negative bacterium, that structurally resemble the AIPs and competitively interfere with AIP binding to AgrC [15,16]. Small molecules also interfere with RNAIII expression but in most cases the mode of action is unknown [17]. However, a group of compounds has recently been found to bind directly to AgrA and interfere with the DNA binding [18,19,20]. Two of these molecules, known as savirin and ω-hydroxyemodin, reduced RNAIII and *psm* expression and reduced infection in a mouse soft tissue model [19,20]. Structurally ω-hydroxyemodin is highly similar to norlichexanthone that we previously isolated from *Penicillium algidum* (IBT 24414) as an inhibitor of *S*. *aureus* virulence gene expression [21]. Xanthones are small, non-reduced tricyclic polyketides occurring in higher plant families, lichen and fungi and they have various biological activities such as being antimicrobial, anti-tumorigenic, antiulcer, and CNS-depressing [22]. Here we aimed to evaluate how norlichexanthone affects virulence gene expression in the serious CA-MRSA strain USA300 and examine its effect on biofilm formation.

## Materials and Methods

### Bacterial strains and growth conditions

*S*. *aureus* strains used in this study were strain 8325–4 [23], Newman [24], FPR3757 (USA300) a multidrug resistant CA-MRSA isolate (obtained from ATCC, Sweden) implicated in outbreaks of skin and soft tissue infection [25], PC322, *hla*::*lacZ* [26], PC203, *spa*::*lacZ* [26], SH101F7, *rnaIII*::*lacZ* [27], and the β-lactamase reporter strain RN10829 [28]. Bacteria were grown in Tryptone Soya Broth (TSB), Oxoid, (1:10 volume/flask ratio), at 37°C with shaking at 200 rpm. Norlichexanthone was purchased from Synthon-Lab Ltd., Bumazhnaya st. 17, office 400, 190020, St-Petersburg, Russia.

### Transcriptional analysis

For RNA isolation cells were inoculated at OD600 = 0.02–0.03. Norlichexanthone was added at OD600 = 0.4. Samples for RNA purification were taken at OD600 = 0.7 and/or 2.0 for strain USA300 and strain Newman, and 1 hour after addition of norlichexanthone for strain 8325–4. Cells were harvested and total RNA was extracted using RNeasy kit (Qiagen) in accordance with the manufacturer’s instructions. RNA quantity and quality were measured on a NanoDrop ND-1000 spectrophotometer by absorbance (A260, A260/A280 and A260/A230 ratio respectively) and by gel electrophoresis. Northern blot analysis was performed as described previously using equal amounts of total RNA for each lane [29]. Probes targeting *hla*, *spa*, RNAIII, *agrA* and *psmα* transcripts were amplified by PCR using the primers (from TAG Copenhagen A/S, Denmark) indicated in Table 1, resulting in probe lengths of 311 bp (*hla*), 719 bp (*spa*), 316 bp (RNAIII), 584 bp (*agrA*) and 176 bp (*psmα*) respectively. For quantification of transcript levels by RT-qPCR residual DNA was removed by treating RNA preparations with DNase I, RNase-Free (Thermo Scientific) according to the manufacturer’s instructions except for an extension of the incubation step to one hour. The RNA was reverse transcribed to cDNA using a High-Capacity cDNA Reverse Transcription Kit (Applied Biosystems), according to the manufacturer’s instructions. To control for DNA contamination, reactions containing no reverse transcriptase were used as negative controls in subsequent amplifications. The cDNA samples were diluted ten-fold and 5 μl was used as template DNA for quantitative PCR of *hla*, *spa*, RNAIII, *saeR*, *lukF*, *sbi* and *coa* using FastStart Essential DNA Green Master and a LightCycler^®^ 96 instrument (both Roche). Results were calculated using the comparative cycle threshold method, in which the amount of target mRNA was normalized to that of the reference genes *ileS* and *pyk*. Reactions were performed in triplicate, and results are presented as the means and standard deviations of the data obtained from 3 biological replicates. Primers used for RT-qPCR and Northern blot analysis are shown in Table 1.

**Table 1:** Primers used in this study.

Gene	Forward primer	Reverse primer
*hla*	CTGTCGCTAATGCCGCAGATTCTG	CTTCTTCGCTATAAACTCTATATTGACCAGC
*spa*	CAAACGGCACTACTGCTGAC	CATGGTTTGCTGGTTGCTTC
RNAIII	GCACTGAGTCCAAGGAAACTAAC	AAGCCATCCCAACTTAATAACC
*saeR*	CCAAGGGAACTCGTTTTACG	GCATAGGGACTTCGTGACC
*lukF*	GCTTATCAGGTGGAGGTAATGG	TGTGCTTCAACATCCCAACC
*sbi*	AAGACAGCAAGAACCCAGACC	CAAACTTGTTGGCTTCTATCAGG
*ileS*	ACATACAGCACCAGGTCACG	CGCCTTCTTCAGTAAATACACC
*pyk*	AGGTTGAACTCCCCAAACAA	GCAGCCCAAGATTACAAAAA
*coa*	ACTAAGGAAGTATACGATCTCGTATCTG	GATTGTCTGTATCTCCAAGGATTAAGTC
*brnQ*	GGACCATTTTTCGCCTTACC	CATGCAATCACAAAGAAGACG
*agrAc*	GTGTACACATATGGATAATAGCGTTGAAACGAT TGAATTAAAACG	GTAGGCACTCGAGTTATATTTTTTTAACGTTTCTCACCGATG
Northern blot primers		
*hla*	GGG TTA GCC TGG CCT TCA GCC	GGG TGC CAT ATA CCG GGT TC
*spa*	GGG GGT GTA GGT ATT GCA TCT G	GGG GCT CCT GAA GGA TCG TC
RNAIII	GGG GAT CAC AGA GAT GTG ATG	GGG CAT AGC ACT GAG TCC AAG G
*agrA*	Ctgataatccttatgaggtgc	cgatgcatagcagtgttc
*psmα*	tatcaaaagcttaatcgaacaattc	ccccttcaaataagatgttcatatc

### Human neutrophil lysis assay

The ability of *S*. *aureus* FPR3757 (USA300) to lyse human neutrophils was tested as described previously [16]. Briefly, sterile filtered supernatants from 7 h and 22.5 h *S*. *aureus* LAC/ FPR3757 (USA300) cultures were grown in 10 mL TSB media/ 100 mL Erlenmeyer flasks, with a start inoculum OD600 = 0.02 and either DMSO (control) or norlichexanthone added to the cultures. Neutrophils were isolated by Ficoll-hypaque purification from fresh heparinized human blood from healthy volunteers according to the regulations of National Committee on Health Research Ethics in Denmark. Blood samples were obtained from volunteer donors by persons not involved in the described analyses according to the regulation of the National Committee on Health research ethics in Denmark (using blood for assessment of effects of microorganisms or microbial components does not require approval from the committee by law no 593 of June 14, 2011). Written consent was obtained from the voluntary donors. Blood was collected for this specific type of analyses. De-identification was done at collection by the person drawing the blood before handing the samples over to the persons performing the analyses.

### *S*. *aureus* aggregation assay

*S*. *aureus* strain 8325–4 was inoculated in TSB (1 mL per well) in a 24-well plate to a start OD600 = 0.02. Norlichexanthone dissolved in DMSO was added from the beginning to a final concentration of 0.5, 1, 5 and 10 μg/mL. No compound and DMSO were used as controls. The plates were incubated 8½ hours at 37°C, with gentle shaking. To quantify the results, the cultures were allowed to sediment for 30 min and the supernatants collected for OD determination at 600 nm, as a lower supernatant OD is indicative of aggregation while single cells remain in suspension increasing the OD. Statistical analysis was performed using the student’s t-test.

### Static biofilm assay

The assay was performed as described [30] with minor modifications. In brief, a starter culture of strain 8325–4 was grown in TSB to OD600 = 0.5. From this culture 50 μL was withdrawn and diluted 10-fold in a 0.9% NaCl solution. 5 μL were inoculated into wells of a 96-well microtiter plate containing 200 μl TSB. Norlichexanthone was added to final concentrations of 0, 0.5, 1, 5 and 10 μg/mL. DMSO and no treatment were used as controls. The plates were incubated for 20 hours at 37°C without shaking. The biofilm was washed twice with (200 μL) 0.9% NaCl, dried and stained with (125 μL) Crystal Violet (0.1%) for 30 min followed by 3x wash with (200 μL) 0.9% NaCl. For quantification purposes the biofilm was solubilized in 96% Ethanol and the absorbance measured at 590 nm using an ELISA plate reader. Statistical analysis was performed using the student’s t-test.

### Activity of norlichexanthone on *P3*::*blaZ* reporter strains

P3 promoter activity was measured in strain RN10829 expressing in trans either wild type AgrC (p*agrC-I-WT*) or a constitutively active variant (pEG11) using a β-lactamase assay as previously described [16]. Briefly, strains were grown in triplicates to OD600 = 0.4–0.5, after which they were split into two cultures and either 5 μg/ml norlichexanthone or DMSO was added. The AgrC-I-WT strain was supplemented with spent medium from strain 8325–4 containing AIP-I at indicated concentrations. Cells were further incubated at 37°C shaking at 200 rpm. At indicated time points OD600 was recorded and samples were withdrawn and frozen immediately (-80°C). β-lactamase activity was determined by adding a final concentration of 0.1 mM nitrocefin (Oxoid) to the samples, and subsequently measuring OD486 over time at 37°C allowing a calculation of arbitrary activity units based on V_max_ (ΔOD486/time) relative to the sample cell density.

### Expression analysis by DNA microarray

*S*. *aureus* strain USA300 was inoculated to an OD600 = 0.02 in Erlenmeyer flasks containing TSB (1/10 flask volume) while shaking at 200 rpm at 37°C. At OD600 = 0.4 either norlichexanthone (to a final concentration of 5 μg/mL) or DMSO (control) was added. Sampling for RNA purification was made at OD600 = 2.0. Biological triplicates were performed. RNA preparation for microarray experiments was carried out using the Agilent FairPlay III kit with Cy3 as the only dye. Arrays were scanned with an Agilent 2-micron scanner and feature data obtained using Agilent's Feature Extraction software. Data were further processed with the Bioconductor software suite [31]. Quantile-base normalization was performed as implemented in the Limma software package [32]. Differential expression was analyzed using Limma (linear models for microarray data) by applying the TREAT methodology [33] to test whether the true differential expression is greater than a given threshold value, (here selected as 1) when examining log2-fold changes.

### Recombinant protein expression and purification of AgrAc

The C-terminal, DNA binding domain of AgrA (AgrAc) from 8325–4 was PCR amplified (using the AgrAc forward and reverse primers) and cloned between the *NdeI* and *XhoI* sites of pET28b to create a recombined plasmid having an in frame fusion of AgrAc with His-tag (Table 1). Expression and purification of AgrAc was carried out as previously described with minor modifications [34]. *E*.*coli* strain BL21 (DE3) strain expressing AgrAc was pre-cultured in 50 ml LB at 37°C overnight. The pre-culture was diluted (1:100) in fresh 1 L LB and cultured at 37°C to OD600 = 0.4–0.6, and then 1 mM of isopropyl β-D-1-thiogalactopyranoside was added. After 20 hours at 16°C, the cells were harvested and lysed in the presence of a protease inhibitor cocktail in a sonicator (8 seconds, stop 5 seconds, 200 cycles). The cell lysate was centrifuged and supernatant was collected (12000 rpm, 30 min at 4°C). Expressed protein was purified from the frozen cells with His-Trap column (Novagen). The purified protein was pooled and loaded onto PD-10 column (GE Healthcare) to remove imidazole and desalted, then was supplemented with 20% glycerol and stored at -80°C.

### Electrophoretic Mobility Shift Assays (EMSA)

The P2-P3 probe (49 bp duplex DNA) covering the *agr* P2 and P3 promoter region was prepared by annealing TTACATTTAACAGTTAAGTATTTATTTCCTCTAGTTAAGCAATATAATG with its complementary oligonucleotide in 25 mM sodium phosphate, 100 mM NaCl, 10 mM DTT at pH 5.8 and purified by size exclusion chromatography using a Superdex 75 column. The DNA probe was mixed with recombined AgrAc (100 μM) in 10 μL reaction mixture consisting of 10 mM Tris·HCl (pH 7.4), 50 mM KCl, 5 mM MgCl_2_, 0.5 μg poly (dI-dC) and either compound or DMSO loading control. After 20 min incubation at 25°C, the samples were mixed with 10 μL of the loading buffer mixture consisting of 10 mM Tris·HCl (pH 7.4), 50 mM KCl, 5 mM MgCl_2_, 20% glycerol. Samples including AgrAc, DNA probe, vehicle and/or compound (0~100 μg/mL) were loaded in TBE buffer containing 10 mM dithiothreitol. The mixtures were analyzed by 4.5% native polyacrylamide gel electrophoresis (120 V for 60 min for pre-run, 10 mA for 20 min, 80 V for 40 min for sample separation) in 1x TBE buffer. The gels were stained with 2 μL SYBR gold (life technologies) in 50 mL 1x TBE buffer. The digital images were acquired and quantified using a phosphor screen (Dark Reader transilluminator DR-88X).

## Results and Discussion

### Norlichexanthone modulates RNAIII, *hla* and *spa* expression

**Fig 1:**
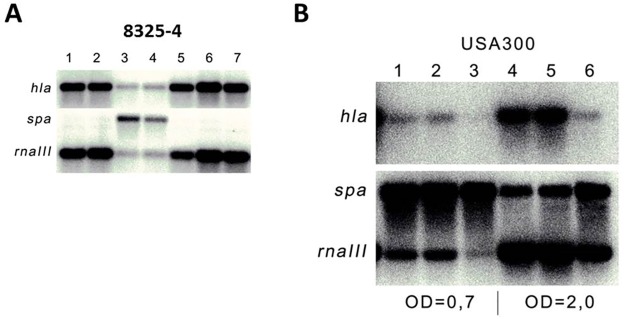
Norlichexanthone affects expression of *spa*, *hla* and RNAIII. Expression of *hla*, *spa* and RNAIII was examined by Northern blot analysis with probes recognizing the transcripts in strain 8325–4 (A) or USA300 (B) treated with in A): 1: No treatment, 2: DMSO (negative control), 3: Norlichexanthone 50 μg/mL (dissolved in DMSO), 4: Norlichexanthone 5 μg/mL, 5: Norlichexanthone 0.5 μg/mL, 6: Norlichexanthone 0.05 μg/mL, 7: Norlichexanthone 0.005 μg/mL added at OD600 = 0.4, and sampled after 1 hour. B) Norlichexanthone 5 μg/mL or DMSO or nothing was added at OD600 = 0.4. RNA was purified from samples taken at OD600 = 0.7 (lanes 1–3) and 2.0 (lanes 4–6). 1: no treatment, 2: DMSO, 3: Norlichexanthone 5 μg/mL, 4: no treatment, 5: DMSO, 6: Norlichexanthone 5 μg/mL.

Previously we used transcriptional reporter gene fusions to show that norlichexanthone extracted from *Penicillium algidum* decreases *hla* and RNAIII expression while enhancing the expression of *spa* in the laboratory strain 8325–4 [21]. When examined by Northern blot analysis we confirmed these findings for both strain 8325–4 (Fig 1A) and the CA-MRSA, USA300 (Fig 1B) with the increase in *spa* transcription being prominent in 8325–4. The expressional changes observed upon norlichexanthone treatment were verified by RT-qPCR (S1 Fig). Approximately 5 μg/mL is required to modulate virulence gene expression (Fig 1A) and at this concentration, growth of *S*. *aureus* is essentially unaffected by the compound (data not shown).

### Norlichexanthone interferes with agr downstream of AgrC activation by AIP, most likely by binding to AgrA

**Fig 2:**
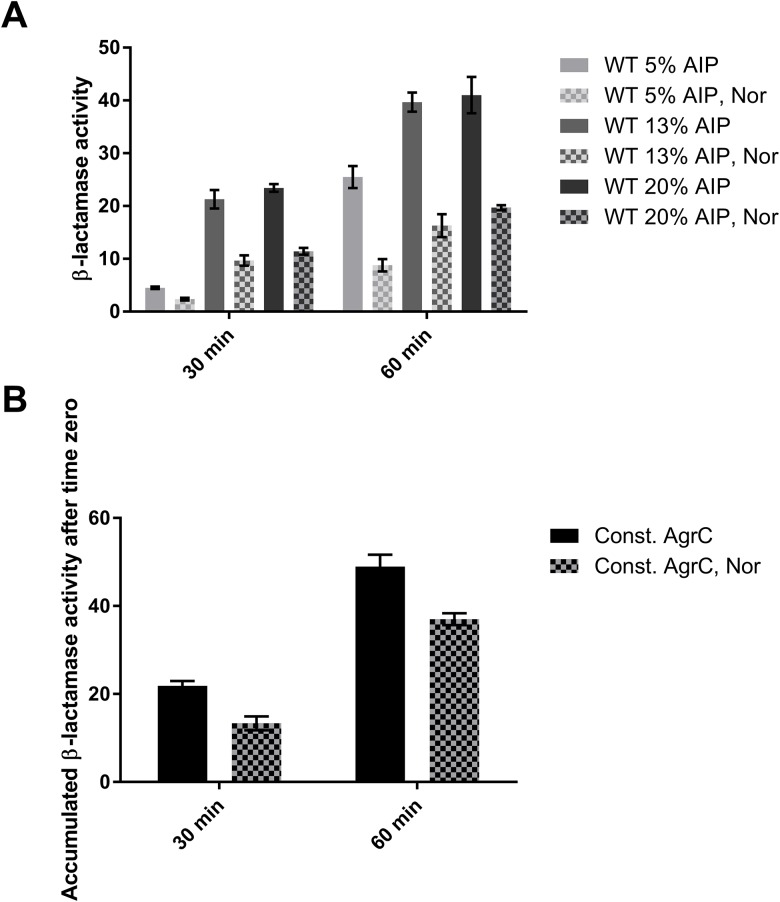
Norlichexanthone reduces RNAIII expression in an AIP concentration-independent manner and also in a mutant with a constitutively active AgrC receptor. (A) Activity of the P3*-blaZ* promoter in a strain expressing a wild type, AIP-inducible AgrC receptor in presence or absence of 5 μg/ml norlichexanthone (Nor). The data represent the mean and standard deviation of the β-lactamase activity obtained from 3 biological replicates at different AIP concentrations (5, 13, and 20%) at time points 30 and 60 minutes. In all conditions, the effect of norlichexanthone is statistically significant (*P*<0.001, two-tailed student’s t-test). (B) Accumulated activity of the P3*-blaZ* promoter in a strain expressing a constitutively active AgrC receptor in presence or absence of 5 μg/ml norlichexanthone (Nor). The β-lactamase activity was measured at 30 and 60 minutes and subtracted the BlaZ activity present already at time 0 to infer the effect on P3 expression after compound addition. The data represent the mean and standard deviation from 3 biological replicates with the effect of norlichexanthone being statistically significant (*P*<0.01 at both time points, two-tailed student’s t-test).

Structurally, norlichexanthone is almost identical to ω-hydroxyemodin and bears some resemblance to savirin (S2 Fig) both of which were demonstrated to bind directly to the DNA binding domain of AgrA [19,20]. We thus hypothesized that due to the structural similarities of norlichexanthone with these two compounds, norlichexanthone could also be binding to AgrA and thus functioning downstream of the AgrC receptor. To support this hypothesis, we employed a reporter strain in which the P3 promoter that normally drives RNAIII expression has been fused to the *blaZ* gene and is uncoupled from activation by the natural AIP-AgrC interaction [16]. In this strain the genes encoding the AIP (*agrB* and *agrD*) have been deleted and thus the strain relies on externally added AIPs. Corroborating the previous transcriptional data, we observed a significant repression of RNAIII promoter activity (approximately 50%) by norlichexanthone in a strain expressing a wild type AgrC that requires AIP addition for activity (Fig 2A). Importantly, we also noted that the relative repression appeared to be independent of the AIP concentration used, which is contrary to what was observed for the competitive inhibitor, solonamide B [16], thus implying that the repression is not linked to the AgrC-AIP interaction Corroborating this interpretation, norlichexanthone is also able to significantly reduce RNAIII promoter activity in a strain expressing a constitutively active AgrC receptor (Fig 2B) ranging from 25–40% at the two time points assayed. These results suggest that norlichexanthone represses the *agr* regulon downstream of AgrC and possibly via binding to AgrA.

**Fig 3:**
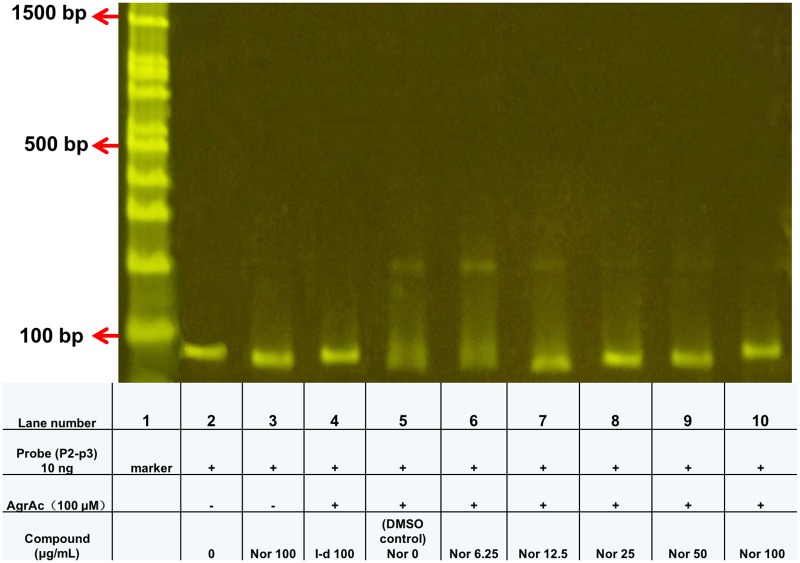
Norlichexanthone interferes with AgrA binding to DNA. Samples including AgrAc, DNA probe, and/or compound were loaded in TBE buffer containing 10 mM dithiothreitol. Assays including the P2-P3 49 bp probe were analyzed in 4.5% native polyacrylamide gels. Lanes: 1. Size marker; 2. P2-P3 probe alone; 3. Probe and norlichexanthone (Nor) in the absence of AgrAc protein; 4. 100 uM I-d (potential hit compound and positive binding control); 5–10: norlichexanthone (Nor) increased by two-fold concentrations from 0–100 μg/ml.

To experimentally address the possibility that norlichexanthone interferes with AgrA binding to DNA, we expressed the C-terminal DNA binding domain of AgrA (AgrAc) and examined its effect on AgrAc binding to the P2-P3 probe carrying AgrA binding sites using the electrophoretic mobility shift assay (EMSA) (Fig 3). The result shows that norlichexanthone at concentrations higher than 12.5 μg/ml prevents AgrA from binding to a fragment containing the binding sites present in the *agr* P2 and P3 region. This data therefore confirms our initial hypothesis that norlichexanthone interferes with *agr*-controlled virulence gene expression via inhibition of AgrA binding to the *agr* P2 and P3 promoter region.

### Norlichexanthone reduces *S*. *aureus* toxicity against human neutrophils

**Fig 4:**
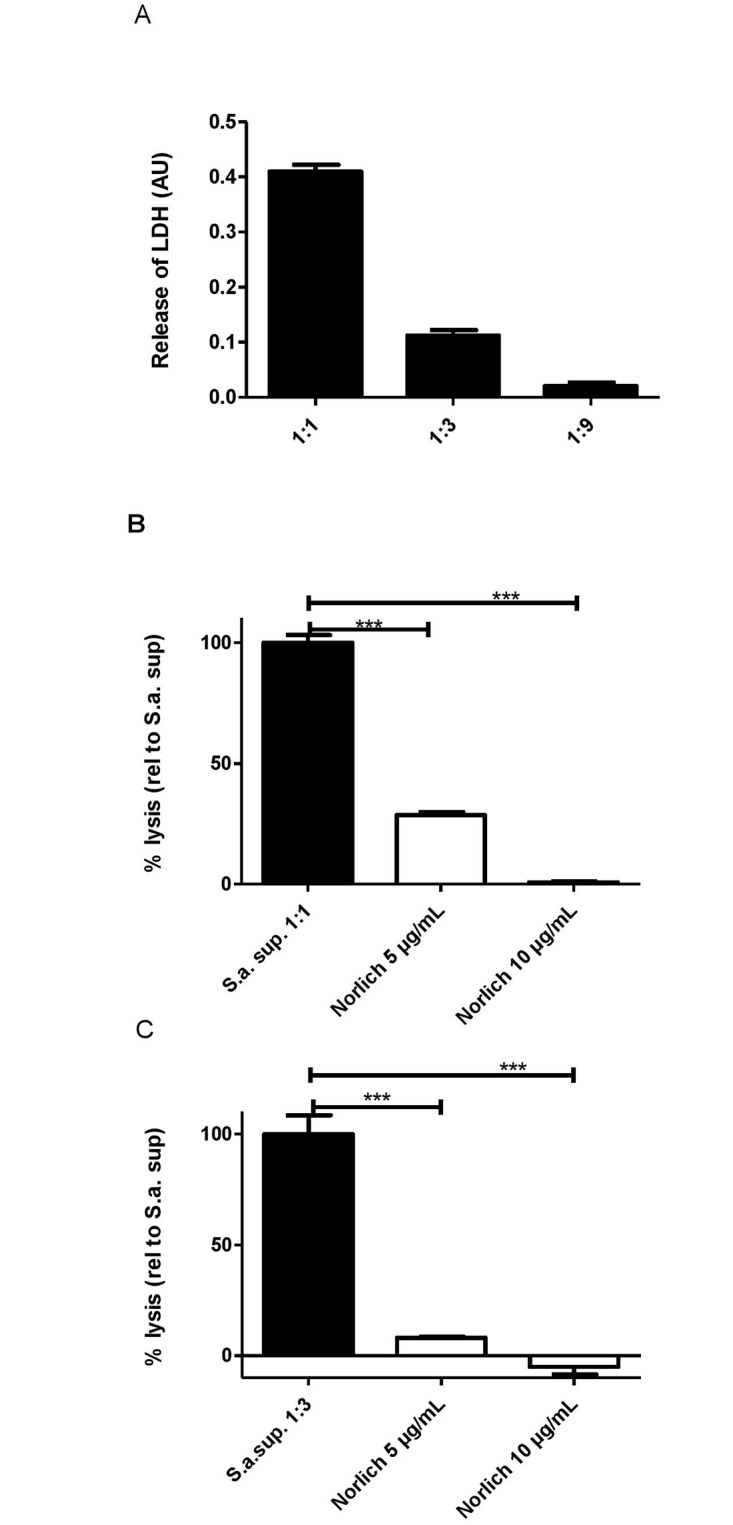
Norlichexanthone protects against *S*. *aureus* mediated neutrophil lysis. Sterile filtered supernatants of *S*. *aureus* USA300 (S.a.) cultures grown for 7 hours with either norlichexanthone at 5 μg/mL or 10 μg/mL or DMSO (control) were added to isolated human neutrophils. The control supernatant where only DMSO was added was examined in 1-fold, 3-fold and 9-fold dilutions (A). Undiluted (1:1) supernatants from norlichexanthone (norlic) treated cultures are shown together with untreated supernatant in (B) and 3-fold dilutions (1:3) are shown in (C). Lysis was monitored by lactate dehydrogenase (LDH) release. Data represents 3 independent experiments, using the average of triplicate wells from each experiment. Asterisk indicates norlichexanthone treated cultures resulting in lysis statistically significant from the corresponding control by one-way ANOVA with Dunnett’s posttest. *, p<0.05, **, p<0.01; ***, p<0.001.

Human neutrophils are one of the primary defences against *S*. *aureus* infections and particularly CA-MRSA strains such as USA300 are adept at inducing rapid neutrophil lysis [35,36]. Neutrophil lysis is mediated in part by the PSMs whose expression is controlled by AgrA [37,38]. To address if norlichexanthone affects S. *aureus* mediated neutrophil lysis we examined culture supernatants of USA300 grown either in the presence or absence of compound. As shown in Fig 4, norlichexanthone reduced the ability of USA300 to lyse human neutrophils when applied at a concentration that reduces *hla* and RNAIII expression. Correlating with the reduced neutrophil lysis of norlichexanthone treated cells, we observed that norlichexanthone treatment also reduced *agrA* expression but only in the exponential growth phase (OD600 = 0.7) and did not significantly affect *psm-α* expression at OD600 = 2.0 (S3 Fig). Thus, norlichexanthone reduced *S*. *aureus* mediated neutrophil lysis and the effect is likely mediated by other toxins than the PSM-α.

### Norlichexanthone reduces biofilm formation and aggregation

**Fig 5:**
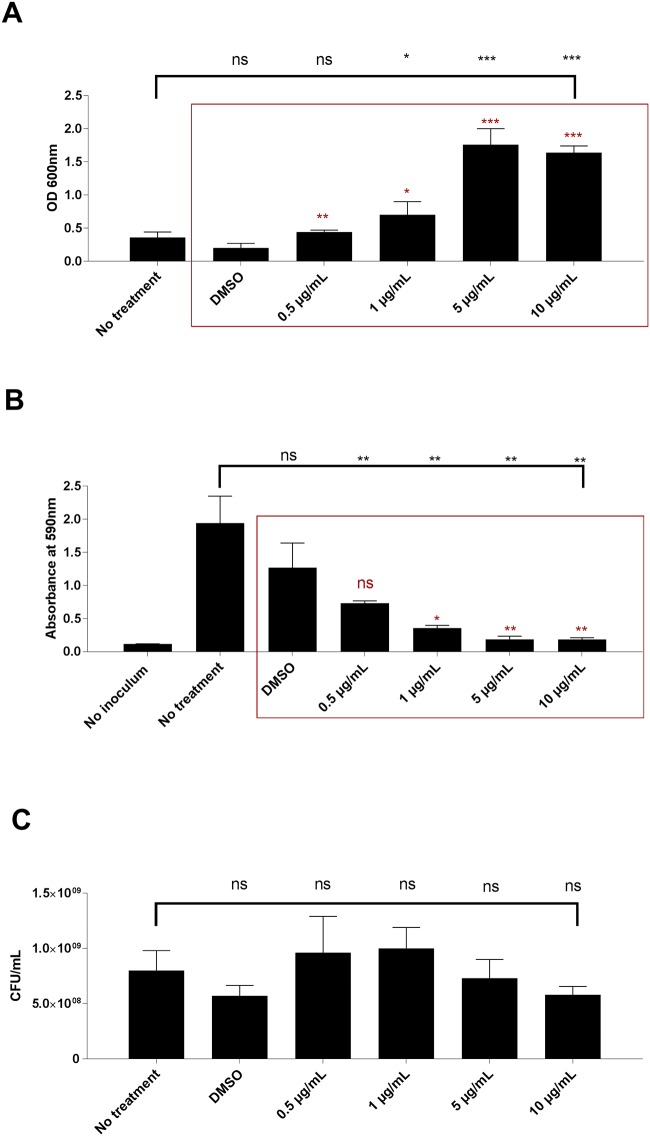
Aggregation and biofilm formation are significantly reduced by norlichexanthone. Strain 8325–4 was cultivated in the presence of DMSO, 0.5, 1, 5 μg/mL or 10 μg/mL norlichexanthone, and incubated 8½ hours at 37°C. (A) Quantification of aggregation. After sedimentation, the supernatants of the cultures were measured for optical density at 600 nm. (B) Dilutions were inoculated into a 96-well microtiter plate containing 200 μL TSB. Norlichexanthone was added to a final concentration of 0.5, 1, 5 and 10 μg/mL. No inoculate, no treatment and DMSO (compound vehicle) were used as controls. The plate was incubated for 20 hours at 37°C without shaking. Biofilm formation was quantified by 0.1% crystal violet staining and OD measurement at 590 nm. (C) Culture CFUs were obtained to eliminate possibility of compound toxicity attributing to reduction of aggregation. Each bar represents the average of 3 replicates and the error bars represent the standard deviation.

The success of *S*. *aureus* as a pathogen is in part attributed to its ability to form biofilm and colonize biotic and abiotic surfaces. Aggregation of unattached cells also appears to be important and in both biofilm and aggregation, antibiotic therapy is highly inefficient [39–41]. To determine if norlichexanthone influences biofilm formation and aggregation we assayed these properties in strain 8325–4 that forms biofilm and is prone to aggregation [39,42]. In the presence of 5 μg/ml norlichexanthone both aggregation (Fig 5A) and biofilm formation (Fig 5B) were greatly reduced while being unaffected by the solvent, DMSO. Furthermore, no CFU reduction was observed (Fig 5C) indicating that these results were not due to compound toxicity, but rather a direct effect on aggregation and biofilm properties. Among the factors that influence biofilm formation is *agr* [43]. However, as *agr* dysfunction generally is associated with increased biofilm formation [43,44] our data indicate that interaction with another regulatory pathway than *agr* is mediating the effect of norlichexanthone on biofilm formation.

### The Sae regulon is repressed by norlichexanthone

In an effort to better understand the effect of norlichexanthone on *S*. *aureus* also with respect to the above results, we analyzed global gene expression in strain USA300 incubated in the presence of 5 μg/ml norlichexanthone for 2 h until an OD600 of 2.0 was reached. Using 3 biological replicates we found 219 genes to be more than 2-fold affected (122 genes with increased expression and 97 genes with decreased expression) compared to the DMSO-treated control cells (S1 Table). Among the genes most prominently affected were *gltAB*, *ilvABCDH*, *sirABC*, *leuABCD*, *metEHNX*, *oppABCDF*, *dapABH*, *ggt*, *hisABDGH*, *thrBC*, *isdACDEF*, *seg*, *srtB*, *hsdMRS*, *and lysC*, which were all more than 2-fold up-regulated; and *hla*, *hlb*, *hlgC*, *fib*, *map*, *clfA*, *lukS*, *sbi*, *epiEFG*, *lip2*, *carA*, *pyrBCEFP*, *sasA*, *saeRS*, *lukF*, *sspAB*, *aur*, *phoP*, *psmα1234* and *arlR* which were all more than 2-fold down-regulated.

Interestingly, among the genes affected by norlichexanthone is a group that is regulated by the two component transcriptional regulatory system, SaeRS namely *lukF*, *sbi*, *hlgC*, *fib*, *hla*, *hlb*, and *saeRS* [44–46] and another group under the control of the stringent response regulator, CodY (*gltAB*, *ilvABCDH*, *leuBCD*, *hisABDGH*, *pyrBCEFP*, *dapABH*, *lysC*, *oppABCDF*) [47]. Sae has been implicated as a key downstream regulator of virulence gene expression in *S*. *aureus* controlling genes being involved in toxicity, immune evasion and bacterial cell adhesion and even biofilm formation [11,14]. While we were unable to verify the effect of norlichexanthone on the CodY regulated genes, the effect on the SaeRS regulated genes was confirmed by quantitative RT-PCR with primers recognizing *saeR*, *lukF* and *sbi* of the SaeRS-regulon, and *brnQ* giving the log2 values of -3.6±0.4; -2.3±0.4; -3.4±0.3; -1.8±0.3 respectively for the indicated genes. These results show that in the presence of norlichexanthone, the SaeRS regulated genes are significantly repressed.

**Fig 6:**
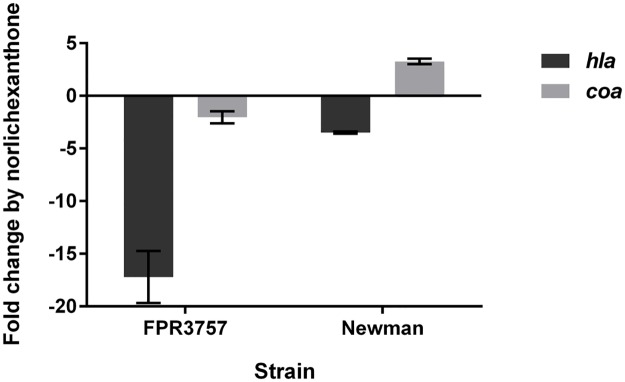
Expression of *coa* and *hla* monitored by quantitative RT-PCR. Expression was monitored in strains USA300 (FPR3757) and Newman by quantitative RT-PCR. Depicted are fold-changes of the transcripts observed upon treatment with 5 μg/ml norlichexanthone relative to DMSO from samples taken at OD600 = 2.0. The data represent the mean and standard deviation from 3 biological replicates.

To further implicate the SaeRS system in response to norlichexanthone we examined the effect of the compound in strain Newman that naturally harbors a mutation in the *saeS*. This mutation allows constitutively active expression of some SaeRS controlled genes including *coa* encoding coagulase, while leaving other SaeRS controlled genes such as *hla* unaffected by the mutation [46]. When examining expression of *coa* and *hla* by qPCR in the presence or absence of norlichexanthone we observed that norlichexanthone caused a reduction in *hla* expression in both strains (although not to the same extent) while *coa* expression was reduced by the compound in USA300 but not in strain Newman (Fig 6). This result together with the microarray data, supports that norlichexanthone may be affecting the Sae system either directly or indirectly. However, as norlichexanthone did not alter expression of protease genes as deduced from the DNA microarray analysis our results do not support a Sae mediated effect on biofilm formation [14].

## Conclusion

Our results show that norlichexanthone reduces virulence gene expression in the CA-MRSA strain USA300 and that it modulates virulence gene expression by interfering with at least two different key virulence gene regulons; the *agr* and the SaeRS two component systems. We successfully show that reduction in RNAIII expression, and thus RNAIII-controlled virulence factors, is a result of direct interaction of norlichexanthone with AgrA, thus blocking AgrA's ability to bind to the P2-P3 promoter region of the *agr* locus. Our microarray data reveal a substantial decrease in *sae* controlled genes in norlichexanthone exposed USA300, implicating a role in SaeRS inhibition. We also show that norlichexanthone not only reduces neutrophil lysis by *S*. *aureus*, but also inhibits both biofilm formation and aggregation, all of which are central to *S*. *aureus*'s success as a human pathogen. Although the exact mechanisms involved in the inhibition of these key virulence traits by norlichexanthone remain to be ascertained, we have nevertheless demonstrated that a small molecule can be an efficient repressor of virulence gene expression and biofilm formation. We thus postulate that such compounds with dual actions can be of particular interest when seeking new treatment options for *S*. *aureus* related infections in the future.

## Supporting Information

S1 Fig Expressional changes of RNAIII, *hla* and *spa* conferred by norlichexanthone. Strain 8325–4 was grown exponentially to OD600 = 0.4 where either 5 μg/mL norlichexanthone or DMSO was added. Transcript levels after 1 hour treatment were determined by RT-qPCR. Depicted mean fold-changes (+/- standard deviation) by norlichexanthone are based upon 3 biological replicates.
(TIF)

S2 Fig Structure of nolirchexanthone, ω-hydroxyemodin and savarin. (TIF)

S3 Fig Norlichexanthone reduces *psm*α and *agr*A expression. Strain USA300 were grown exponentially to OD600 = 0.4 where either 5 μg/mL norlichexanthone (nor), DMSO or nothing was added. RNA was purified from samples collected at OD600 = 0.7 and 2.0, and analyzed by Northern blotting where equal amounts of RNA was loaded. The membrane was probed with radioactive labeled probes targeting *agr*A and *psm*α.
(TIF)

S1 Table Transcriptome analysis of USA300 exposed to norlichexanthone. Cells were grown in TSB and treated with 5 μg/mL norlichexanthone or corresponding amount of DMSO at OD600 = 0.4. Data is based on biological triplicates, sampled at OD600 = 2.0. Fold regulation indicates norlichexanthone treated cells relative to DMSO treated cells.
(DOCX)
