# Supplementary figures and images for: Norlichexanthone Reduces Virulence Gene Expression and Biofilm Formation in Staphylococcus aureus
Source: PLoS One. 2016 Dec 22;11(12):e0168305. doi: 10.1371/journal.pone.0168305 (PMC5179057; doi:10.1371/journal.pone.0168305)

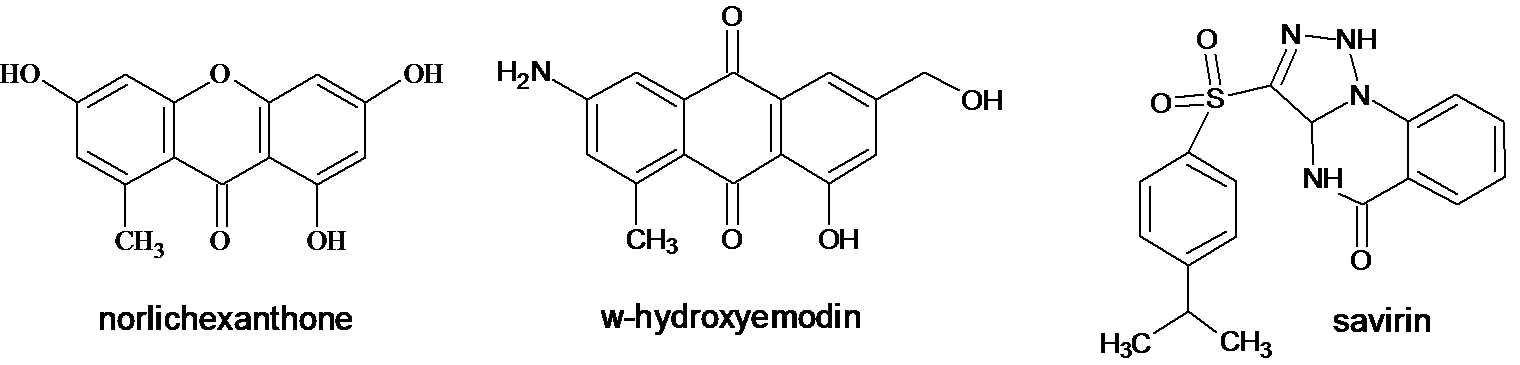

Supplement: S2 Fig — (TIF) [file pone.0168305.s002.tif]

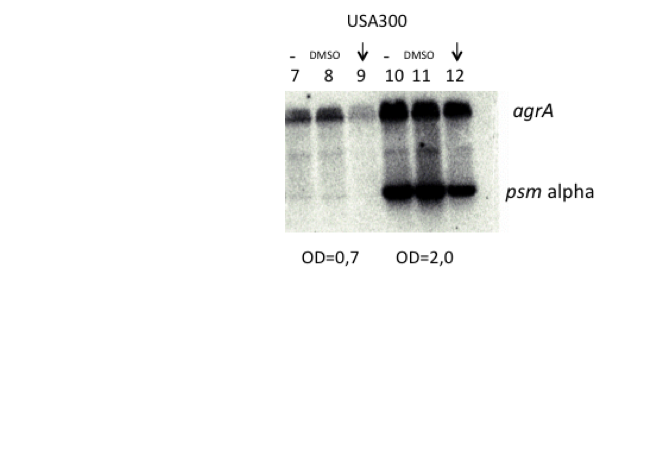

Supplement: S3 Fig — Strain USA300 were grown exponentially to OD600 = 0.4 where either 5 μg/mL norlichexanthone (nor), DMSO or nothing was added. RNA was purified from samples collected at OD600 = 0.7 and 2.0, and analyzed by Northern blotting where equal amounts of RNA was loaded. The membrane was probed with radioactive labeled probes targeting agrA and psmα. (TIF) [file pone.0168305.s003.tif]
