# Supplementary material for: Norlichexanthone Reduces Virulence Gene Expression and Biofilm Formation in Staphylococcus aureus
Source: PLoS One. 2016 Dec 22;11(12):e0168305. doi: 10.1371/journal.pone.0168305 (PMC5179057; doi:10.1371/journal.pone.0168305)
Supplement: S1 Table — Cells were grown in TSB and treated with 5 μg/mL norlichexanthone or corresponding amount of DMSO at OD600 = 0.4. Data is based on biological triplicates, sampled at OD600 = 2.0. Fold regulation indicates norlichexanthone treated cells relative to DMSO treated cells. (DOCX) [file pone.0168305.s004.docx]

**S1 Table**

| Fold regulation | GeneName | Description | P.Value | SAUSA300 (FPR3757) annotation |
| --- | --- | --- | --- | --- |
| 13,45 |  | Putative membrane protein | 0,038 | 2321 |
| 10,7 | gltA | Glutamate synthase, large subunit | 0,07 | 445 |
| 9 | gltB | Glutamate synthase, small subunit | 0,059 | 446 |
| 7,9 |  | ABC transporter ATP-binding protein | 0,037 | 2453 |
| 6,8 | ilvH |  | 0,044 | 2008 |
| 6,4 |  | Putative membrane protein | 0,023 | 2507 |
| 5,9 |  | Putative transport system membrane protein | 0,071 | 436 |
| 5,3 | ilvB | Acetolactate synthase | 0,053 | 2007 |
| 5,3 |  | Putative membrane protein | 0,107 | 2454 |
| 5,2 | ilvC | Ketol-acid reductoisomerase | 0,068 | 2009 |
| 5,2 |  | Amino acid permease family protein | 0,024 | 2538 |
| 5 | ilvD | Dihydroxy-acid dehydratase | 0,021 | 2006 |
| 4,9 | sirA | Lipoprotein | 0,09 | 117 |
| 4,7 | sirC | Iron-regulated ABC transporter siderophore permease protein SirC | 0,086 | 115 |
| 4,6 | metN1 | Methionine import ATP-binding protein metN 1 | 0,067 | 435 |
| 4,6 | leuA | 2-isopropylmalate synthase | 0,073 | 2010 |
| 4,6 |  | Putative uncharacterised protein SA2143 | 0,059 | 2299 |
| 4,3 | oppD | Putative oligopeptide transport ATP-binding protein | 0,053 | 889 |
| 4,2 |  | Putative lipoprotein | 0,073 | 437 |
| 4,2 |  | Putative ABC transporter ATP-binding protein SAR2766 | 0,049 | 2617 |
| 4 | oppF | Putative oligopeptide transport ATP-binding protein | 0,067 | 890 |
| 4 |  | Putative uncharacterised protein | 0,047 | 2619 |
| 3,9 | leuB | 3-isopropylmalate dehydrogenase | 0,05 | 2011 |
| 3,9 | leuC | 3-isopropylmalate dehydratase large subunit | 0,096 | 2012 |
| 3,8 | leuD | 3-isopropylmalate dehydratase small subunit | 0,102 | 2013 |
| 3,8 | ilvA | Threonine dehydratase biosynthetic | 0,014 | 2014 |
| 3,7 | oppC | Putative oligopeptide transport system permease protein | 0,055 | 888 |
| 3,6 |  | Putative lipoprotein | 0,046 | 798 |
| 3,6 | oppA | Transport system extracellular binding lipoprotein | 0,091 | 891 |
| 3,5 | sirB | Putative siderophore transport system permease | 0,154 | 116 |
| 3,5 |  | ABC transporter permease protein | 0,089 | 797 |
| 3,5 |  | UPF0397 protein SAR2767 | 0,098 | 2618 |
| 3,4 |  | Putative transport system permease | 0,114 | 201 |
| 3,4 | dapB | Dihydrodipicolinate reductase | 0,061 | 1289 |
| 3,3 |  | Putative membrane protein | 0,048 | 11 |
| 3,3 |  | Putative transport protein | 0,061 | 2298 |
| 3,2 | oppB | Putative oligopeptide transport system permease protein | 0,099 | 887 |
| 3,2 |  | Transport system extracellular binding lipoprotein | 0,104 | 891 |
| 3,1 |  | Cys/Met metabolism PLP-dependent enzyme | 0,074 | 359 |
| 3,1 |  | ABC transporter permease protein | 0,068 | 2616 |
| 3 |  | Putative membrane protein | 0,045 | 230 |
| 3 |  | Cys/Met metabolism PLP-dependent enzyme | 0,069 | 360 |
| 3 | metN2 | Methionine import ATP-binding protein metN 2 | 0,066 | 796 |
| 3 | dapA | Dihydrodipicolinate synthase | 0,076 | 1288 |
| 3 |  | Putative aminotransferase | 0,054 | 2539 |
| 3 | hisG | ATP phosphoribosyltransferase | 0,039 | 2612 |
| 3 |  | Putative lipoprotein | 0,058 | 2614 |
| 3 |  | Putative uncharacterised protein | 0,082 | 2615 |
| 2,9 |  | SA0010 protein | 0,063 | 10 |
| 2,9 | adhC | Alcohol dehydrogenase, zinc-containing | 0,076 | 55 |
| 2,9 | rlp | RGD-containing lipoprotein | 0,158 | 203 |
| 2,9 | ggt | Putative gamma-glutamyltranspeptidase | 0,118 | 204 |
| 2,8 |  | Putative uncharacterised protein SA0887 | 0,001 | 935 |
| 2,8 | opp-1D | Oligopeptide transporter putative ATPase domain | 0,075 | 2408 |
| 2,7 |  | Prophage L54a, HNH endonuclease family protein | 0,064 | 1406 |
| 2,6 |  | Putative hydrolase | 0,061 | 12 |
| 2,6 |  | ABC transporter ATP-binding protein | 0,039 | 200 |
| 2,6 |  | Sodium: alanine symporter family protein | 0,067 | 914 |
| 2,6 |  | Homoserine dehydrogenase | 0,076 | 1226 |
| 2,6 | asd | Aspartate semialdehyde dehydrogenase | 0,123 | 1287 |
| 2,6 | dapH | 2,3,4,5-tetrahydropyridine-2,6-dicarboxylate N-acetyltransferase | 0,082 | 1290 |
| 2,6 |  | D-3-phosphoglycerate dehydrogenase | 0,125 | 1670 |
| 2,5 |  | Putative transport system permease | 0,236 | 202 |
| 2,5 | metE | 5-methyltetrahydropteroyltriglutamate--homocysteine methyltransferase | 0,107 | 357 |
| 2,5 | pdxS | Pyridoxal biosynthesis lyase pdxS | 0,051 | 504 |
| 2,5 |  | Putative uncharacterised protein | 0,002 | 929 |
| 2,5 | isdC | Iron-regulated surface determinant protein C | 0,07 | 1030 |
| 2,5 | thrB | Homoserine kinase | 0,075 | 1228 |
| 2,5 |  | Putative uncharacterised protein | 0,057 | 1706 |
| 2,5 |  | Putative aminotransferase | 0,027 | 2610 |
| 2,4 |  | SA1979 protein | 0,113 | 2136 |
| 2,4 | hisH | Imidazole glycerol phosphate synthase subunit hisH | 0,036 | 2608 |
| 2,35 |  | FecCD transport family protein | 0,145 | 2135 |
| 2,3 |  | Putative lipoprotein | 0,217 | 175 |
| 2,3 |  | Methylenetetrahydrofolate reductase | 0,079 | 358 |
| 2,3 |  | Type I restriction modification system modification protein | 0,058 | 405 |
| 2,3 | hsdS | Probable restriction modification system specificity subunit | 0,096 | 406 |
| 2,3 |  | Putative uncharacterised protein | 0,024 | 553 |
| 2,3 | srtB | Sortase B | 0,064 | 1034 |
| 2,3 | lysC | Aspartokinase | 0,078 | 1286 |
| 2,3 |  | Putative uncharacterised protein | 0,083 | 2086 |
| 2,3 |  | Putative peptidase | 0,032 | 2087 |
| 2,3 | opp-1B | Oligopeptide transporter putative membrane permease | 0,057 | 2410 |
| 2,3 | sasG | Putative surface protein SA2285 | 0,097 | 2436 |
| 2,2 | hmp | Flavohemoprotein | 0,064 | 234 |
| 2,2 |  | Uncharacterised protein SAR0806 | 0,023 | 736 |
| 2,2 |  | Putative membrane protein | 0,042 | 934 |
| 2,2 | isdA | Iron-regulated surface determinant protein A | 0,043 | 1029 |
| 2,2 | isdD | Putative membrane protein | 0,067 | 1031 |
| 2,2 | isdE | High-affinity heme uptake system protein isdE | 0,158 | 1032 |
| 2,2 |  | Putative soluble hydrogenase subunit | 0,153 | 1669 |
| 2,2 |  | Putative uncharacterised protein | 0,02 | 1903 |
| 2,2 | opp-1C | Oligopeptide transporter putative membrane permease | 0,101 | 2409 |
| 2,2 | opp-1A | Oligopeptide transporter putative substrate binding domain | 0,075 | 2411 |
| 2,2 |  | Putative transport protein | 0,132 | 2417 |
| 2,2 |  | Uncharacterised protein SAR2570 | 0,044 | 2424 |
| 2,2 | hisA | 1-(5-phosphoribosyl)-5-[(5-phosphoribosylamino)methylideneamino] imidazole-4-carboxamide isomerase | 0,014 | 2607 |
| 2,1 | dnaC | DnaB-like helicase | 0,117 | 16 |
| 2,1 |  | Putative ABC transporter ATP-binding protein | 0,339 | 174 |
| 2,1 |  | Putative branched-chain amino acid transport system carrier protein | 0,104 | 188 |
| 2,1 | hsdR | Type-1 restriction enzyme R protein | 0,05 | 196 |
| 2,1 | pdxT | Glutamine amidotransferase subunit pdxT | 0,126 | 505 |
| 2,1 | sstA | SA0688 protein | 0,041 | 718 |
| 2,1 |  | Putative lipoprotein | 0,099 | 992 |
| 2,1 | isdF | Probable heme-iron transport system permease protein isdF | 0,083 | 1033 |
| 2,1 | thrC | Threonine synthase | 0,183 | 1227 |
| 2,1 |  | Haloacid dehalogenase-like hydrolase | 0,07 | 1229 |
| 2,1 |  | Putative uncharacterised protein | 0,195 | 1997 |
| 2,1 |  | Putative membrane protein | 0,12 | 1998 |
| 2,1 |  | Putative membrane protein | 0,131 | 2139 |
| 2,1 |  | Putative exported protein | 0,104 | 2493 |
| 2,1 | hisB | Imidazoleglycerol-phosphate dehydratase | 0,113 | 2609 |
| 2,1 | hisD | Histidinol dehydrogenase | 0,015 | 2611 |
| 2 |  | Cysteine synthase | 0,12 | 118 |
| 2 |  | Putative ornithine cyclodeaminase | 0,088 | 119 |
| 2 |  | Galactosyl transferase | 0,059 | 132 |
| 2 |  | Putative acetyltransferase | 0,056 | 343 |
| 2 |  | Uncharacterised hydrolase SAR1410 | 0,129 | 1291 |
| 2 |  |  | 0,144 | 2268 |
| 2 |  | Uncharacterised lipoprotein SA2275 | 0,097 | 2430 |
| 2 |  | D-isomer specific 2-hydroxyacid dehydrogenase | 0,08 | 2496 |
| 2 |  | Putative uncharacterised protein SA2484 | 0,064 | 2625 |
| -2 | rbsD | D-ribose pyranase | 0,095 | 263 |
| -2 | rbsU | Putative ribose uptake protein rbsU | 0,107 | 264 |
| -2 | essC | Protein essC | 0,038 | 283 |
| -2 | esxB | Virulence factor esxB | 0,071 | 285 |
| -2 | mnhA2 | Putative antiporter subunit mnhA2 | 0,177 | 610 |
| -2 |  | Putative uncharacterised protein | 0,131 | 794 |
| -2 | sdhB | Putative succinate dehydrogenase iron-sulfur protein | 0,081 | 1048 |
| -2 |  | Putative uncharacterised protein | 0,097 | 1099 |
| -2 | arlR | Response regulator arlR | 0,261 | 1308 |
| -2 |  | Lipoprotein, putative | 0,008 | 1440 |
| -2 | pckA | Phosphoenolpyruvate carboxykinase [ATP] | 0,146 | 1731 |
| -2 | hlb | Hypothetical phage protein | 0,013 | 1937 |
| -2 |  | Single-stranded DNA-binding protein | 0,184 | 2052 |
| -2 | mtlF | Mannitol-specific phosphotransferase enzyme IIA component | 0,08 | 2107 |
| -2 | ureE | Urease accessory protein ureE | 0,052 | 2241 |
| -2 |  | Putative membrane protein | 0,072 | 2448 |
| -2 | clpL | ATP-dependent Clp protease ATP-binding subunit clpL | 0,074 | 2486 |
| -2 |  | Putative uncharacterised protein SA2372 | 0,122 | 2523 |
| -2,1 |  | RpiR family transcriptional regulator | 0,046 | 195 |
| -2,1 | essA | Protein essA | 0,023 | 280 |
| -2,1 |  | Putative uncharacterised protein SA0280 | 0,067 | 287 |
| -2,1 | psmA1 | Phenol-soluble modulin alpha 1 peptide | 0,14 | 424 |
| -2,1 | pstB | Phosphate import ATP-binding protein pstB | 0,023 | 1280 |
| -2,1 | pstS | Phosphate-binding protein pstS | 0,116 | 1283 |
| -2,1 |  | Putative uncharacterised protein SA1320 | 0,075 | 1439 |
| -2,1 |  | Putative lipoprotein | 0,115 | 1739 |
| -2,1 |  | Putative transcriptional antiterminator | 0,107 | 2106 |
| -2,1 | fabG2 | SA2260 protein | 0,084 | 2416 |
| -2,1 | lip1 | Lipase 1 | 0,056 | 2603 |
| -2,15 |  | Putative uncharacterised protein SA0279 | 0,044 | 286 |
| -2,2 | scdA | Cell wall-related protein scdA | 0,204 | 253 |
| -2,2 | lrgA | Antiholin-like protein lrgA | 0,205 | 256 |
| -2,2 | lrgB | Antiholin-like protein lrgB | 0,199 | 257 |
| -2,2 | sspA | Glutamyl endopeptidase | 0,086 | 951 |
| -2,2 | pyrB | Aspartate carbamoyltransferase | 0,034 | 1093 |
| -2,2 | phoP | Alkaline phosphatase synthesis transcriptional regulatory protein | 0,196 | 1639 |
| -2,2 |  | Putative uncharacterised protein | 0,077 | 1750 |
| -2,2 | epiE | Epidermin immunity protein F | 0,148 | 1761 |
| -2,2 |  | Putative uncharacterised protein | 0,116 | 2446 |
| -2,2 | aur | Zinc metalloproteinase aureolysin | 0,114 | 2572 |
| -2,3 |  | Putative ribokinase | 0,034 | 262 |
| -2,3 | esaC | Protein esaC | 0,039 | 284 |
| -2,3 | lpl4nm | Uncharacterised lipoprotein SAUSA300_0414 | 0,117 | 414 |
| -2,3 | saeR | Response regulator saeR | 0,057 | 691 |
| -2,3 | comFA | Putative helicase | 0,067 | 734 |
| -2,3 | epiG | Epidermin immunity protein F | 0,157 | 1760 |
| -2,3 | hlb | Holin | 0,012 | 1924 |
| -2,3 | hlgC | Gamma-hemolysin component C | 0,071 | 2366 |
| -2,3 |  | Putative uncharacterised protein | 0,174 | 2522 |
| -2,3 |  | Putative uncharacterised protein | 0,062 | 2524 |
| -2,3 | sraP | Serine-rich adhesin for platelets | 0,048 | 2589 |
| -2,4 |  | Putative uncharacterised protein SA0576 | 0,139 | 608 |
| -2,4 |  | Putative thioredoxin | 0,114 | 795 |
| -2,4 | pyrP | Putative uracil permease | 0,012 | 1092 |
| -2,4 | lukF | Panton-Valentine leukocidin chain F | 0,04 | 1381 |
| -2,4 |  | Conserved domain protein, putative | 0,035 | 1740 |
| -2,4 | truncated(hlb) | Truncated beta-hemolysin | 0,157 | 1918 |
| -2,5 | saeS | Histidine protein kinase saeS | 0,054 | 690 |
| -2,5 | sspB | Staphopain B | 0,014 | 950 |
| -2,5 | malR | Maltose operon transcriptional repressor | 0,184 | 1457 |
| -2,5 |  | Putative uncharacterised protein | 0,012 | 2334 |
| -2,5 |  | Putative PTS transport system, IIABC component | 0,097 | 2576 |
| -2,6 |  | Putative uncharacterised protein | 0,139 | 192 |
| -2,6 | fruA | Fructose specific permease | 0,08 | 685 |
| -2,6 |  | Putative regulatory protein | 0,039 | 928 |
| -2,6 |  | Putative exported protein | 0,132 | 1052 |
| -2,6 | pyrE | Orotate phosphoribosyltransferase | 0,076 | 1098 |
| -2,6 | odhA | 2-oxoglutarate dehydrogenase E1 component | 0,071 | 1306 |
| -2,6 |  | Terminase small subunit | 0,022 | 1405 |
| -2,6 |  | Putative uncharacterised protein | 0,029 | 2415 |
| -2,7 | lip2 | Lipase 2 | 0,045 | 320 |
| -2,7 | pyrC | Dihydroorotase | 0,015 | 1094 |
| -2,7 | carA | Carbamoyl-phosphate synthase small chain | 0,054 | 1095 |
| -2,7 | pyrF | Orotidine 5'-phosphate decarboxylase | 0,03 | 1097 |
| -2,7 | lukS | Panton-Valentine leukocidin chain S | 0,031 | 1382 |
| -2,7 | malA | Alpha-D-1,4-glucosidase | 0,108 | 1456 |
| -2,7 |  | Putative terminase small subunit | 0,087 | 1981 |
| -2,8 | epiF | Epidermin immunity protein F | 0,07 | 1762 |
| -2,8 | sbi | Immunoglobulin-binding protein sbi | 0,027 | 2364 |
| -2,9 | murQ | N-acetylmuramic acid 6-phosphate etherase | 0,058 | 193 |
| -2,9 |  | DeoR ramily regulatory protein | 0,102 | 683 |
| -2,9 |  | Putative phosphofructokinase | 0,088 | 684 |
| -2,9 | lukF | Uncharacterised leukocidin-like protein 1 | 0,199 | 1974 |
| -2,9 | arcA | Arginine deiminase | 0,011 | 2570 |
| -3 | clfA | Clumping factor A | 0,082 | 772 |
| -3 | truncated-SA | Putative uncharacterised protein | 0,24 | 793 |
| -3,1 | lukS | Uncharacterised leukocidin-like protein 2 | 0,146 | 1975 |
| -3,3 | carB | Carbamoyl-phosphate synthase large chain | 0,027 | 1096 |
| -3,6 | map | 77 kDa membrane protein | 0,235 | 1869 |
| -3,8 | fib | Fibrinogen-binding protein | 0,146 | 1055 |
| -4 |  | Putative membrane protein | 0,026 | 692 |
| -4,4 |  | Putative exported protein | 0,134 | 409 |
| -5,1 |  | Putative membrane protein | 0,032 | 692 |
| -5,4 |  | Surface protein, putative | 0,105 | 2164 |
| -5,7 |  | Putative lipoprotein | 0,051 | 693 |
| -6 |  | SA1004 protein | 0,044 | 1056 |
| -7,8 | hla | Alpha-Hemolysin | 0,051 | 1058 |
